# Assessing the latest advances in bone marrow stem cell therapy for Avascular Necrosis hip: A comprehensive systematic review, meta-analysis, and meta-regression of randomized controlled trial studies

**DOI:** 10.1371/journal.pone.0297319

**Published:** 2025-06-24

**Authors:** Robin Novriansyah, Tanti Ajoe Kesoema, Kevin Christian Tjandra, I. Nyoman Sebastian Sudiasa, Shakira Amirah, Imke Maria Del Rosario Puling, Prudence Lucianus, Revina Maharani, Danendra Rakha Putra Respati, Laksmana Adi Krista Nugraha, Ismail Hadisoebroto Dilogo

**Affiliations:** 1 Department of Surgery, Faculty of Medicine, Universitas Diponegoro, Semarang, Indonesia; 2 Kariadi General Hospital, Semarang, Indonesia; 3 Department of Physical Medicine and Rehabilitation, Faculty of Medicine, Diponegoro University, Semarang, Indonesia; 4 Department of Medicine, Faculty of Medicine, Universitas Diponegoro, Semarang, Indonesia; 5 Faculty of Medicine, Universitas Indonesia, Jakarta, Indonesia; 6 Department of Medicine, Faculty of Medicine, Universitas Brawijaya,; 7 Stem Cell Medical Technology Integrated Service Unit, Cipto Mangunkusumo Central Hospital, Faculty of Medicine, Universitas Indonesia, Jakarta, Indonesia; 8 Stem Cell and Tissue Engineering Research Cluster, Indonesian Medical Education and Research Institute (IMERI), Universitas Indonesia, Jakarta, Indonesia; 9 Department of Orthopaedic and Traumatology, Cipto Mangunkusumo General Hospital, Faculty of Medicine, Universitas Indonesia, Jakarta, Indonesia; Nanjing Medical University, CHINA

## Abstract

**Background:**

Avascular necrosis (AVN) of the hip is a disease characterized by vascular disruptions, where 67% of untreated cases may lead to the collapse of the femoral head. None of the current approaches, such as core decompression (CD), vascularized bone grafting, osteotomy, tissue implantation, and other methods, have been proven fully effective in delaying the progression of osteonecrosis. Recent findings indicate that bone marrow stem cells (BMSCs) have significant potential to regenerate necrotic tissue and prevent the progression of AVN in the hip. This study aims to evaluate the efficacy, side effects, treatment failure rate, most effective treatment stage of AVN hip, and application technique to treat AVN hip.

**Method:**

We performed this systematic review and meta-analysis from randomized controlled trials (RCTs) and sources published between 2013 and 2023 from six databases. The literature searching method was based on predetermined PICOS, study eligibility criteria, and PRISMA guidelines. The extracted data were then assessed quantitatively using R Studio with Harris hip score (HHS), Visual analog scale (VAS), the collapse of the femoral head, and conversion of total hip arthroplasty (THA) as the outcomes of interest, then qualitatively using RoB Tool, the extracted data were analyzed using R Studio.

**Result:**

A total of 12,939 records were identified through database searching. After the removal of duplicates and non-randomized studies using automation tools, 4,846 articles were screened. Following title and abstract review, 10 studies met the eligibility criteria and were included in the systematic review and meta-analysis, comprising 593 patients and 779 hips.

The included studies originated from China (n = 4), France (n = 1), South Korea (n = 1), Australia (n = 1), Iran (n = 1), India (n = 1), and Spain (n = 1). Risk of bias assessment using RoB 2.0 tool revealed 70% of the RCTs were rated as having a low risk of bias, while 30% were judged to have some concerns.

Meta-analysis demonstrated that BMSC therapy significantly reduced the risk of femoral head collapse (OR = 0.15; 95% CI: 0.09–0.25; P < 0.00001; I² = 0%) and conversion to THA (OR = 0.20; 95% CI: 0.13–0.31; P < 0.00001; I² = 83%). Functional outcomes, as measured by the HHS, were significantly improved in the BMSC group (MD = 10.70; 95% CI: 9.70–11.69; P < 0.00001; I² = 51%). Pain reduction, assessed using the VAS, also favored BMSC therapy (MD = −8.04; 95% CI: −8.66 to −7.42; P < 0.00001; I² = 99%).

Meta-regression analyses explored the influence of study-level covariates on outcomes. No predictor reached statistical significance for HHS or THA conversion. However, mean age showed a borderline significant association with VAS (coefficient = −0.8029; P = 0.065), suggesting a possible trend of more significant pain reduction in older patients.

**Conclusion:**

Currently established AVN hip treatments are proven to be less effective. The findings strongly support that giving a BMSC at the early-stage AVN hip could improve the patient’s clinical outcome and have fewer side effects and treatment failure compared to conventional treatment.

## Introduction

Avascular necrosis of the hip, also known as osteonecrosis of the hip, is a debilitating condition characterized by the disruption of blood supply to the femoral head, leading to the death of osteocytes and bone marrow [1,2]. This vascular disruption often results in the collapse of the femoral head if left untreated, with an annual incidence of 10,000–20,000 new cases reported in the United States. This condition is more prevalent in males than females [1,3].

The bilateral involvement of AVN is common, with approximately 72% of patients developing the condition in the second hip within two years after the first hip is affected [4]. Various treatment options, such as CD, non-vascularized bone grafting, porous tantalum implants, and vascularized bone grafting, have been explored. However, none of these treatments have proven to be fully effective in halting the progression of AVN [4,5].

Current surgical approaches, including CD, vascularized bone grafting, osteotomies, tissue-engineered material implantation, and THA, have shown limited success in delaying the progression of AVN. As a result, there is a growing interest in exploring alternative treatments, such as stem cell therapy, which has shown promise in regenerative medicine for various conditions [6–8].

Recent studies have highlighted a significant reduction in the quantity of endothelial progenitor cells and colony-forming units in patients with AVN of the hip, suggesting a role for stem cells in the disease’s pathogenesis and treatment [9]. Mesenchymal stem cells (MSCs), in particular, have demonstrated the potential to differentiate into osteoblasts, chondrocytes, and adipocytes, offering a regenerative solution to counteract the effects of AVN [9,10]. A meta-analysis by Mao et al. (2020) provided strong evidence supporting the long-term benefits of stem cell therapy in preventing the progression of early-stage osteonecrosis of the femoral head (ONFH) [11,12].

Given these findings, this study aims to assess the efficacy of stem cell therapy in patients with AVN of the hip at various stages. The study will evaluate outcomes using the VAS, HHS, conversion to THA, and the occurrence of femoral head collapse. Additionally, this study seeks to identify the optimal dose of BMSCs and determine the most effective application technique.

## Materials and methods

### Registration

The present systematic review employed the Preferred Reporting Items for Systematic Reviews and Meta-Analyses (PRISMA) guidelines. On December 3, 2023, the registration of this systematic review and meta-analysis was recorded on the Open Science Framework (OSF) under the following DOI: https://doi.org/10.17605/OSF.IO/DHFM4.

### Eligibility criteria

This systematic review and meta-analysis incorporated original studies published between 2013 and 2023, with the final search conducted on September 18, 2023. The study design inclusion criteria encompassed RCTs or clinical trials specifically addressing the treatment of AVN utilizing BMSC. Exclusion criteria comprised technical reports, editorial responses, narrative reviews, systematic reviews, meta-analyses, non-comparative research, in silico studies, in vitro studies, in vivo studies, scientific posters, study protocols, and conference abstracts. Additionally, non-English and non-full-text papers unrelated to the application of BMSCs in knee osteoarthritis were excluded.

The selected articles adhered to the PICO criteria, with the following specifications: i) patients diagnosed with hip osteonecrosis; ii) intervention involving the application of BMSCs for the treatment of hip AVN; iii) comparison with standard current treatment or CD without the use of BMSCs; and iv) outcomes assessed encompassed the evaluation of HHS, volume of repair, collapse of the femoral head, conversion to THA, and VAS for pain assessment.

### Outcome measure

The assessment of the therapeutic efficacy in this study involved the evaluation of various outcomes, including the HHS, volume of cartilage repair, femoral head collapse, conversion to THA, and the VAS. The HHS served as a measure to evaluate the hip pathology condition both before and after the intervention. Assessment of cartilage volume repair was conducted pre- and post-treatment to gauge the regeneration of cartilage. Evaluation of femoral head collapse aimed to identify potential complications associated with the treatment. The assessment of THA conversion rates was employed to ascertain the treatment’s failure rate. Additionally, the VAS score was utilized to gauge clinical pain levels before and after the intervention.

### Index test

This systematic review and meta-analysis incorporated studies that furnished data about the application and assessments of bone marrow mesenchymal stem cells in the context of hip AVN.

### Reference standard

In this systematic review and meta-analysis, reference standards were established through the inclusion of professional research utilizing RCTs or clinical trials. These studies were employed to evaluate the impact of BMSCs application on outcomes related to hip AVN.

### Data sources and search

The research articles included in this investigation were sourced from databases such as Scopus, PubMed, Cochrane, Sage Pub, ProQuest, and Science Direct. The search covered a period of 10 years leading up to September 18, 2023, preceding the current review. Boolean operators were applied within the Medical Subject Headings (MeSH) framework, utilizing specific keywords from the National Institute of Health (NIH) National Library of Medicine browser. Table 1 displays the exact search strings and keywords employed in each database. Mendeley Group Reference Manager in the authors’ library was utilized for the organization and retention of the identified studies.

**Table 1:** Keywords Used in Literature Searching.

Database	Keywords
Scopus	AVN AND Hip joint AND Stem cell AND Therapeutic
Pubmed	
Cochrane	
Sage Pub	
Proquest	
Science Direct	

Different article types were used as filters in the respective databases: research articles in Scopus, Cochrane, Sage Pub, and ProQuest; clinical trial and RCTs types in PubMed; content-type articles in the Springer database; and research articles in Science Direct. A total of 12,939 studies were initially retrieved from these databases (5,622 from Scopus, 32 from PubMed, 156 from Cochrane, 116 from Sage Pub, 5,311 from ProQuest, and 1,702 from Science Direct). After applying predetermined inclusion and exclusion criteria, 4,873 studies were imported into the Mendeley Group Reference Manager for further screening.

The search strategy is designed for thoroughness and reproducibility. However, non-English papers and studies related to knee osteoarthritis were excluded to focus on high-quality, relevant studies directly addressing the clinical question. The exclusion of non-English papers was based on the potential for translation inaccuracies and the aim to include studies readily accessible to the global research community. Similarly, studies related to knee osteoarthritis were excluded to avoid confounding results and maintain a clear focus on the intended scope of this review.

### Selection process

The search terms outlined in Table 1 underwent utilization across six databases by six independent reviewers and one validator. Articles underwent scrutiny through the examination of abstracts and full texts to identify and exclude irrelevant articles as well as studies with dissimilar designs. A total of 1,505 studies were excluded for lacking pertinent information concerning the research question (the therapeutic efficacy of bone marrow mesenchymal stem cell treatment in hip AVN patients), and an additional 11 studies were eliminated due to unmatched designs. Subsequently, 3,357 articles underwent screening for retrievable full-text and alignment with the desired outcomes. Of these, 3,330 articles were excluded due to their incongruence with the specified outcomes. A total of 10 studies underwent assessment for risk of bias utilizing Cochrane’s RoB 2 tool. All 10 studies were appraised as possessing good quality, and the selection process was meticulously documented in the PRISMA flow chart. The summary of study selection process was summarized in Supporting File 1

### Data collection process

Data from 10 studies were collected and organized within a Google Spreadsheet, incorporating the following parameters: i) First, author and year; ii) Second, country; iii) third, study design; iv) fourth, sample size; v) fifth, percentage of male hips; vi) sixth, mean age; vii) seventh, disease stage; viii) eighth, disease stage for the intervention group; ix) ninth, diagnostic criteria; x) tenth, intervention utilized; xi) eleventh, amount of mesenchymal stem cells delivered; xii) twelfth, control; xiii) thirteenth, HHS 12 months post-intervention; xiv) fourteenth, volume of repair; xv) fifteenth, collapse of the femoral head; xvi) sixteenth, conversion to THA; xvii) seventeenth, VAS for pain assessment. If missing data could not be retrieved, available data from the publication were used, and variables with unavailable data were recorded as “not reported (NR)” and excluded from specific analyses where applicable. Any discrepancies among reviewers were addressed through comprehensive discussions to achieve consensus. The summary of data extraction was summarized Supporting File 2

### Study risk of bias assessment (qualitative synthesis)

The evaluation of bias in each study included in the analysis was initially conducted independently by RN, TAK, KCT, INSS, SA, IMDRP, PL, RM, DRPR, and LAKN using the Cochrane RoB 2 tool. If discrepancies arose during the assessment, the reviewers first discussed the issue collectively. In situations where consensus could not be reached after discussion, IHD, as the corresponding author, made the final decision. Any study identified as having a high risk of bias was excluded from the analysis. The RoB 2 tool was utilized to assess bias across various domains, including the randomization process, deviations from the intended intervention, missing outcome data, measurement of the outcome, and selection of the reported result. Each domain was individually assessed and categorized as low risk, some concerns, or high risk of bias. The risk of bias assessment process was documented in the Supporting File 3

### Reporting bias assessment

Result from the Cochrane RoB 2 tool showed all 10 articles included did not show any high risk of bias so no articles were excluded. 5 studies showed some concerns of bias, and 5 studies showed low risk of bias.

### Statistical analysis

All meta-analyses were conducted using R studio, employing a common-effects or random-effect model to account for anticipated variability across studies in terms of design, population characteristics, and interventions. This model was selected to better accommodate true differences among studies, beyond random sampling error, which is particularly important in the presence of clinical heterogeneity.

For continuous outcomes, standardized mean differences (SMD) with 95% confidence intervals (CI) were used instead of MD, as the included studies utilized various measurement scales. This standardization ensured comparability across studies. For dichotomous outcomes, we applied the Mantel-Haenszel method to calculate pooled risk ratios during subgroup analyses, offering reliable estimates despite differing sample sizes among studies.

Heterogeneity was assessed using both the I² statistic and Chi-square (χ²) test. I² values were categorized as follows: low (<25%), moderate (25–74%), and high (≥75%). Values exceeding 25% were considered meaningful and guided the decision to apply a random-effects model. For studies reporting medians with interquartile ranges (IQRs) or ranges (minimum–maximum), means and standard deviations were estimated using the transformation methods proposed by Luo et al. and Wan et al. to ensure consistent data presentation and accurate pooling.

Publication bias was evaluated through funnel plot visualization. In cases of funnel plot asymmetry, we examined study characteristics and methodology to distinguish between true publication bias and heterogeneity. Sensitivity analyses were also conducted by including only studies assessed as having a low risk of bias, in order to verify the robustness of the overall findings.

To further investigate sources of heterogeneity (I² > 50%), meta-regression was performed using R Studio. Potential confounding factors included total number of hips, proportion of male and female participants, disease stage, and mean age. This regression analysis aimed to explore how study-level characteristics may have contributed to variability in outcomes.

Additionally, leave-one-out (LOO) sensitivity analysis was performed utilizing R studio to assess the influence of individual studies on the overall pooled effect estimate. In this method, each study was sequentially omitted, and the meta-analysis was re-run to evaluate the stability and robustness of the results against potential outliers or overly influential studies.

## Result

### Study selection

**Fig 1:**
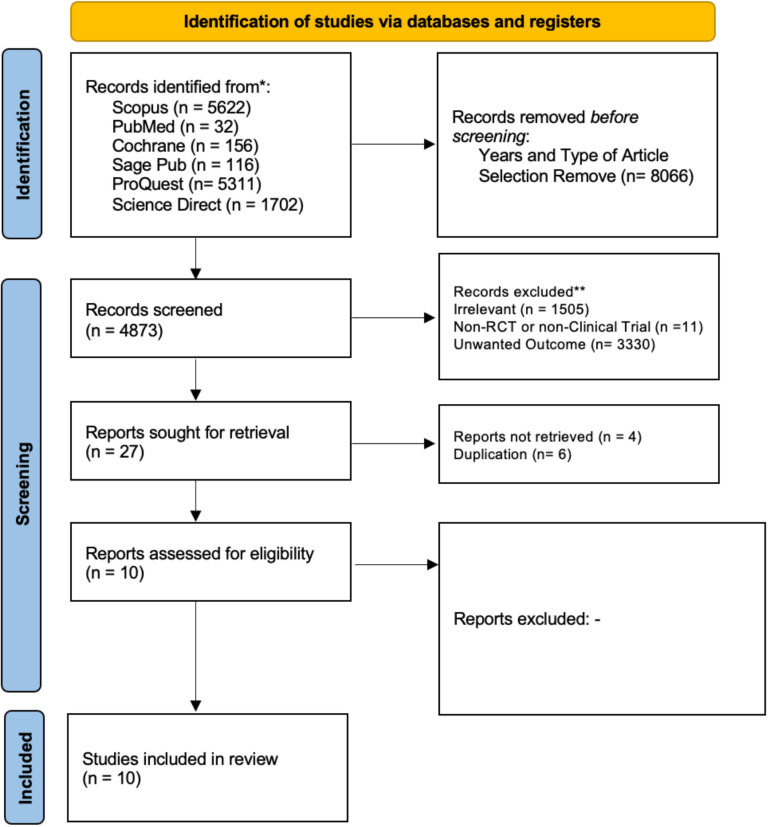
PRISMA 2020 flow diagram.

Overall, 12,939 studies were produced by doing a literature search using six databases: ProQuest, PubMed, Cochrane, Sage Pub, Scopus, and ScienceDirect. In order to exclude non-RCT studies, automation options from each database were employed, which led to the exclusion of 8,066 publications. 4,846 irrelevant topics, design articles, and outcomes were removed after the authors evaluated every 27 articles from the title and abstract for relevance. The complete texts of ten articles were then obtained. Lastly, the author determined the eligibility of each study and all ten articles passed the eligibility criteria. ten papers were included in this systematic review and meta-analysis. Our study selection process is presented in the PRISMA diagram flow chart in Fig 1.

### Study characteristics

In total, 593 samples with 779 number of hips from 10 studies were included in the analysis. 4 studies were conducted in China, 1 study in France, 1 study in South Korea, 1 study in Australia, 1 study in Iran, 1 study in India, and 1 study in Spain. All extracted data were attached in the study characteristic table, Table 2.

**Table 2:** Study Characteristic.

No.	Title	Author; Year	Country/ Place	Study Design	Study Period	Population				
						Sample size (n)	Number of H	Male (%)	Age in years (Mean±SD)	Disease Stage (intervention)
1	Treatment of early stage osteonecrosis of the femoral head with autologous implantation of bone marrow-derived and cultured mesenchymal stem cells	Dewei, et al; 2012 [13]	China	Randomized Clinical Trial	May 2004 – July 2006	100	104	52.00%	Intervention: 33.8 (7.70); Control: 32.7 (10.5)	IC (3); IIA (15); IIB (24); IIC (11);
2	Clinical efficiency of bone marrow mesenchymal stem cell implantation for osteonecrosis of the femoral head: a matched pair control study with simple core decompression	Kang, et al; 2018 [14]	South Korea	Matched Pair Control Study	February 2004 – October 2014	100	106	74.00%	Intervention: 46.0 ± 9.3; Control: 47.3 ± 9.7	I (1); II (29); III (19); IV (4)
3	Cell therapy versus simultaneous contralateral decompression in symptomatic corticosteroid osteonecrosis: a thirty year follow-up prospective randomized study of one hundred and twenty five adult patients	Hernigou, et al; 2018 [15]	France	Randomized Clinical Trial	1988 - 1998	125	250	62.00%	Intervention and Intervention: 36	I (138); II (112);
4	Autologous Bone Marrow Mesenchymal Stem Cells Associated with Tantalum Rod Implantation and Vascularized Iliac Grafting for the Treatment of End-Stage Osteonecrosis of the Femoral Head	Dewei, et al; 2014 [16]	China	Randomized Clinical Trial	October 2007 – October 2008	24	31	54.00%	Intervention and Intervention: 33.21 ± 6.09	IIIC (19); IV(12)
5	Efficacy of autologous bone marrow buffy coat grafting combined with core decompression in patients with avascular necrosis of femoral head: a prospective, double-blinded, randomized, controlled study	Ma et al., 2014 [17]	Australia	Prospective, double-blinded, randomized controlled trial	June 2009 – October 2010	21	49	71.00%	Intervention: 35.60 ± 8.05 Control: 34.78 ± 11.48	I(3); II(17); III(5)
6	Combining Concentrated Autologous Bone Marrow Stem Cells Injection With Core Decompression Improves Outcome for Patients with Early-Stage Osteonecrosis of the Femoral Head: A Comparative Study	Tabatabaee, et al; 2015 [18]	Iran	prospective, randomized study	Not stated	18	28	68.00%	Intervention 31 ± 11.4 Control 26.8 ± 5.8	I (3), II (9), III (2)
7	Early Results of Core Decompression and Autologous Bone Marrow Mononuclear Cells Instillation in Femoral Head Osteonecrosis A Randomized Control Study	Sen, et al; 2012 [19]	India	Randomized Clinical Trial	Not stated	18	18	16.67%	61.8 ± 6.6	Not Stated
8	Autologous mesenchymal stem cell implantation in the management of osteonecrosis of the femoral head	Yan, et al; 2015 [20]	China	Retrospective, Clinical Trial	June 2005 – July 2008	56	56	48.21%	Not Stated	ICRS grade 2 (8); ICRS grade 3 (16); ICRS grade 4 (32)
9	Mid-term comparative outcomes of autologous bone-marrow concentration to treat osteonecrosis of the femoral head in standard practice	Cruz-Pardos, et al; 2016 [21]	Spain	Retrospective, Randomize Study	1999 - 2012	30	30	63.33%	Control: 60.3 Low-dose: 65.9 High-dose: 57.8	Not stated
10	Core decompression and implantation of bone marrow mononuclear cells with porous hydroxyapatite composite filler for the treatment of osteonecrosis of the femoral head	Liu et al; 2013 [22]	China	Retrospective, Clinical Trial	2006 - 2010	34	34	79.41%	Intervention: 38.0 ± 4.9 Control: 38.1 ± 6.1	IIB (25); IIC (30)

### Risk of bias in studies

**Fig 2:**
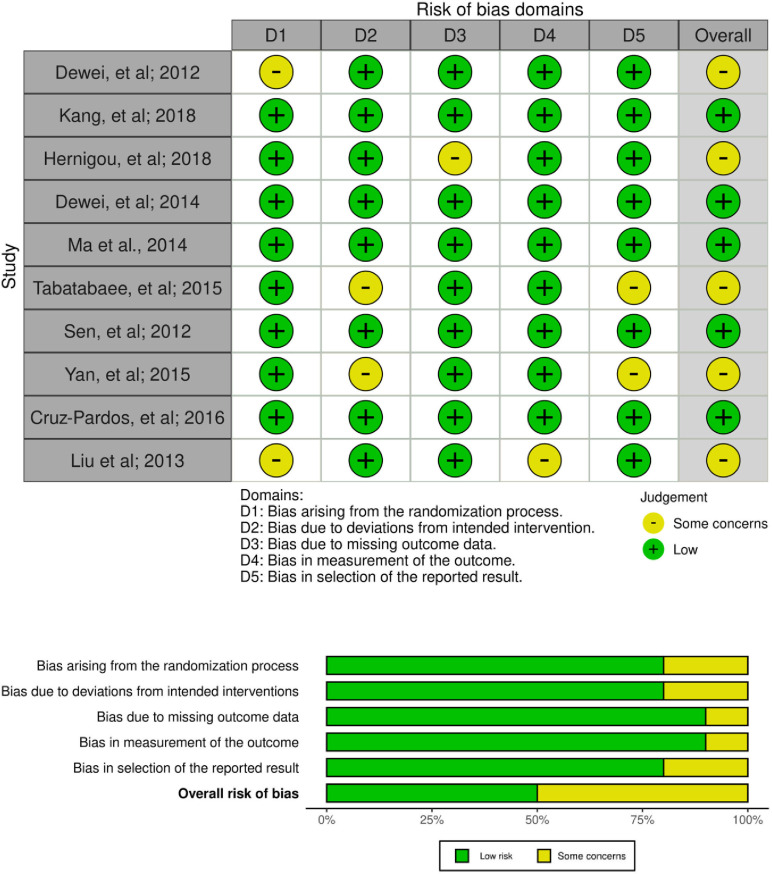
Risk of bias assessment result.

The risk of bias in the included studies was assessed using the Revised Tool for Risk of Bias in Randomized Trials (RoB 2.0) (Fig 2). Based on the evaluation, 70% of the RCTs were rated as having a low risk of bias, while 30% were judged to have some concerns.

Among the ten RCTs evaluated with the RoB 2.0 tool, three studies were rated as having “some concerns,” primarily arising from issues in the randomization process (Domain 1) and missing outcome data (Domain 3). The remaining seven studies were judged to have a low risk of bias across all five assessed domains, including bias arising from the randomization process, deviations from intended interventions, missing outcome data, measurement of the outcome, and selection of the reported result. Overall, the majority of included studies demonstrated a low risk of bias, supporting the credibility and internal validity of the findings (Fig 2).

### Study result summaries

The method employed for journal selection yielded ten studies that were utilized in the systematic review and meta-analysis. These studies authored by Dewei, et al. (2012) [13]; Kang, et al. (2018) [14]; Hernigou et al. (2018) [15]; Dewei, et al. (2014) [16]; Ma, et al. (2014) [17]; Tabatabaee, et al. (2015) [18]; Sen, et al. (2012) [19]; Yan, et al. (2015) [20]; Cruz-Pardos, et al. (2016) [21]; Liu, et al. (2013) [22] were included in the analysis. Brief profiles of these nine studies are summarized in Table 2.

### Collapse of femoral head

**Fig 3:**
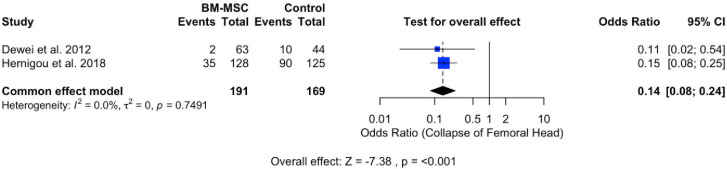
Forest plot of collapse of the femoral head.

The assessment of treatment complications involved the evaluation of the occurrence of femoral head collapse incidents. Two studies analyzing the complication rate were included in the meta-analysis, as depicted in Fig 3. The forest plot revealed a statistically significant effect (P < 0.001), with a pooled odds ratio of 0.14 (95% CI: 0.09, 0.25). The pooled odds ratio indicates a reduced likelihood of complications such as femoral head collapse. Heterogeneity was observed (I^2^ = 0%; P = 0.7491).

### Conversion of total hip arthroplasty (THA)

**Fig 4:**
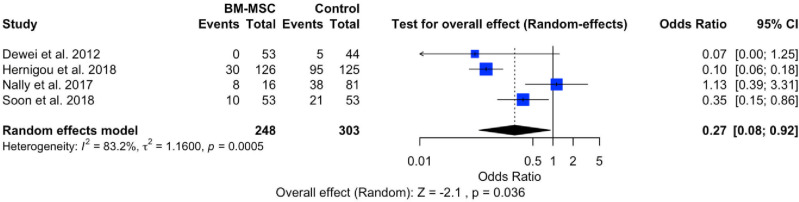
Forest plot of conversion of THA.

The evaluation of treatment failure in hip AVN is based on the events of conversion to THA. The meta-analysis, encompassing four studies related to treatment failure, is illustrated in Fig 4. The forest plot indicates a significant effect within the intervention group (P < 0.0036), with a pooled odds ratio of 0.27 (95% CI: 0.08, 0.92). This odds ratio suggests a lower failure rate in the intervention group compared to the control group. However, the forest plot also reveals the presence of heterogeneity (I^2^ = 83%; P = 0.0005).

### Harris Hip Score (HHS)

**Fig 5:**
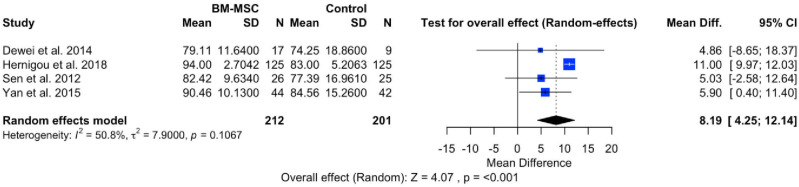
Forest plot of HHS.

The evaluation of hip pathology before and after the intervention was conducted using the HHS, encompassing pain severity, function, absence of deformity, and range of motion in standardized tools, with scores ranging from 0 (worse disability) to 100 (less disability). A meta-analysis of four studies aimed to assess the efficacy of BMSCs in reducing hip disability between pre-intervention and post-intervention groups. The forest plot in Fig 5 illustrates a significant effect (P < 0.001), with a pooled MD of 8.19 (95% CI: 4.25, 12.14). This outcome suggests a significant increase in HHS in patients with AVN hip who received BMSCs treatment. The forest plot also reveals heterogeneity (I^2^ = 50.8%; P = 0.1067).

### Visual Analog Scale (VAS)

**Fig 6:**
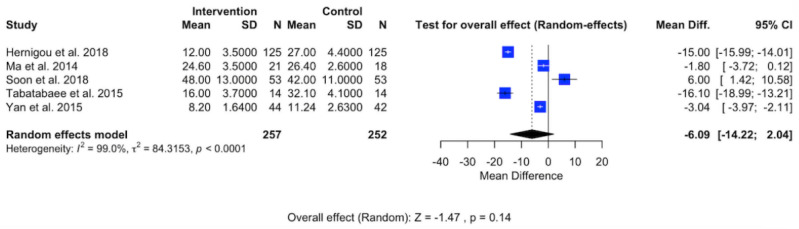
Forest plot of VAS.

Clinical pain experienced by patients with AVN in the hip was assessed using VAS scores, as illustrated in Fig 6. The meta-analysis, inclusive of five studies, aimed to analyze the effectiveness of BMSCs in alleviating clinical pain in AVN hip patients. The forest plot reveals a substantial effect (P < 0.14) with a pooled MD of −6.09 (95% CI: −14.22, −2.04). This pooled MD indicates that mesenchymal stem cell treatment significantly reduces clinical pain in patients with AVN hip. However, the forest plot also indicates a high level of heterogeneity (I^2^ = 99%; P < 0.0001).

### Meta-regression

**Fig 7:**
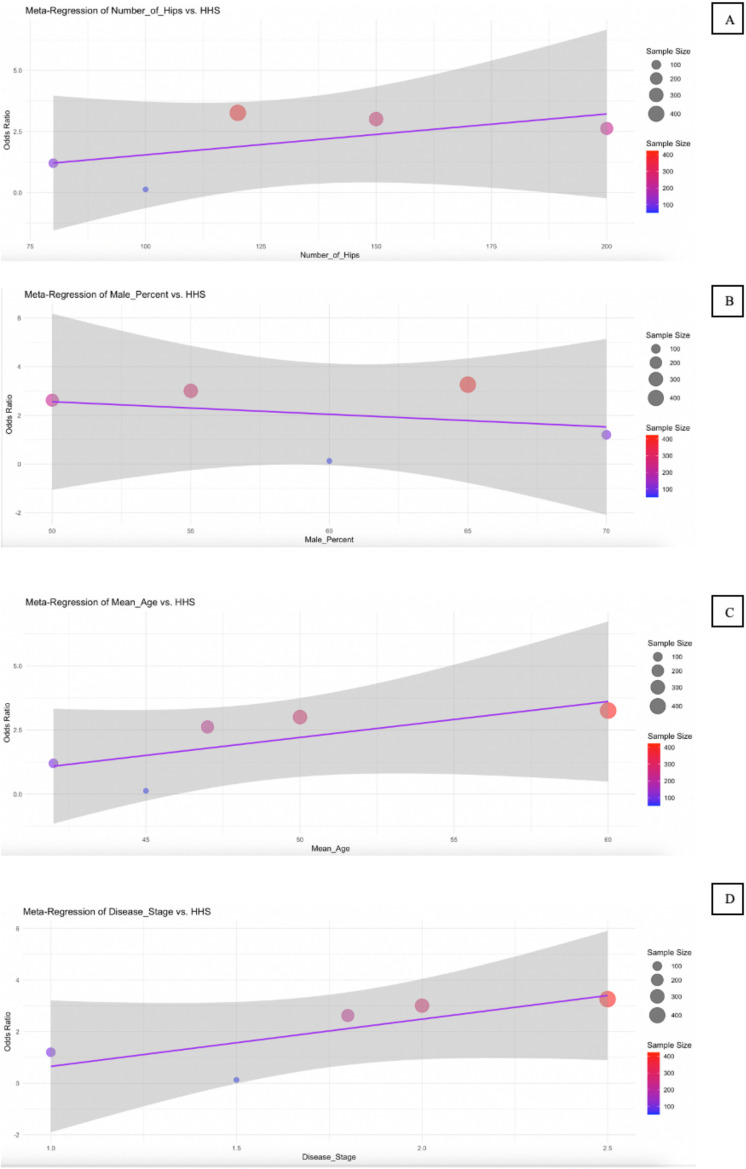
Bubble plot for meta-regression of HHS. Meta-regression analyzes the effect of Number of Hips (A), Male Percent (B), Mean Age (C), and Disease Stage (D) on the HHS.

**Fig 8:**
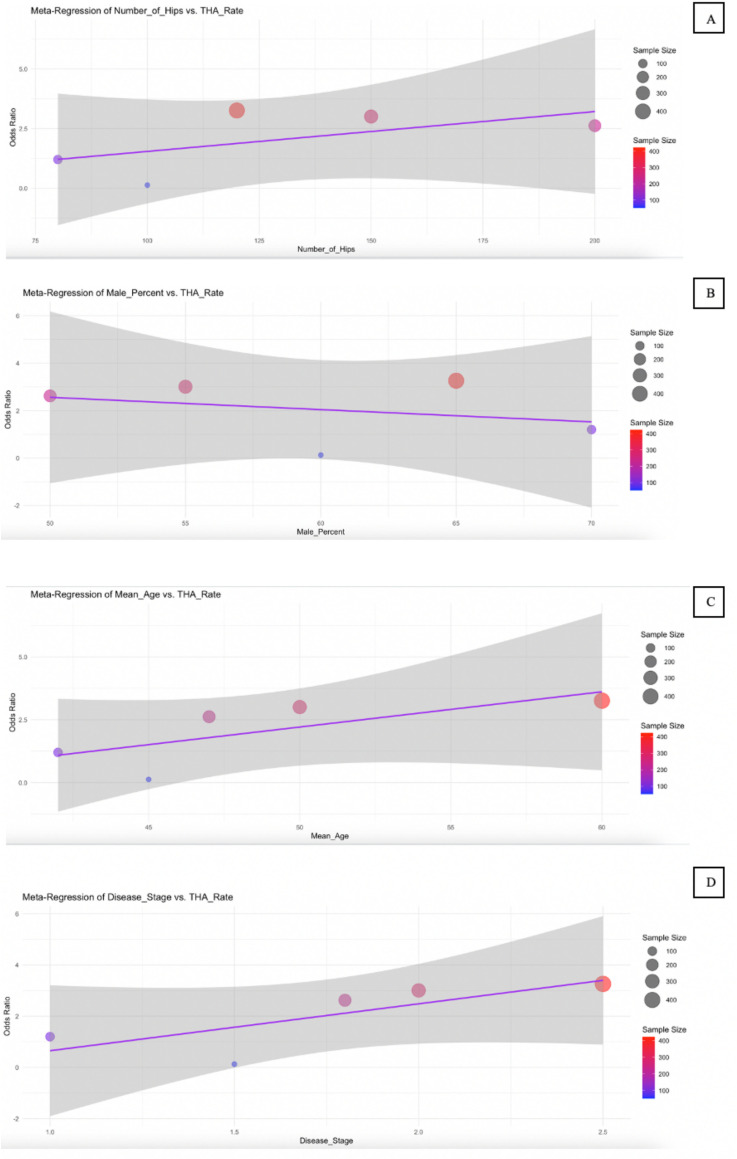
Bubble plot for meta-regression of Conversion of THA. Meta-regression analyzes the effect of Number of Hips (A), Male Percent (B), Mean Age (C), and Disease Stage (D) on the Conversion of THA.

**Fig 9:**
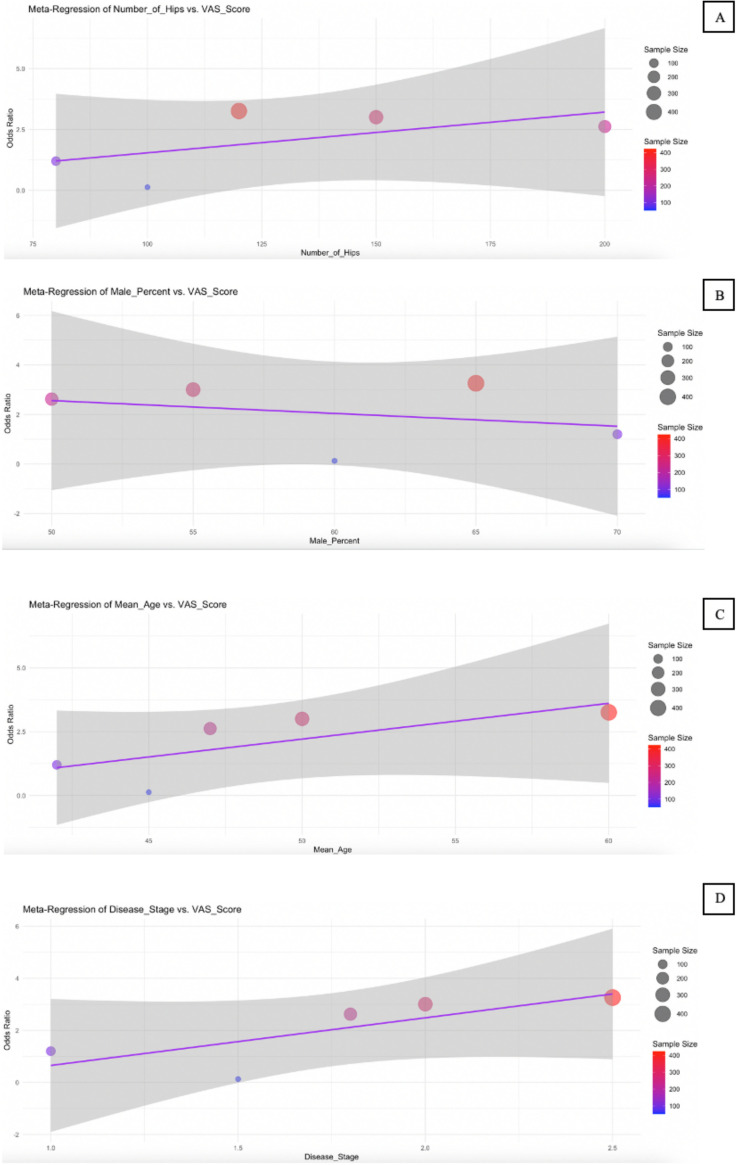
Bubble plot for meta-regression of VAS Score. Meta-regression analyzes the effect of the Number of Hips (A), Male Percent (B), Mean Age (C), and Disease Stage (D) on the VAS Score.

The meta-regression results presented in Table 3 and illustrated in Figs 7 to 9 explore the influence of several study-level predictors—number of hips, male percentage, mean age, and disease stage—on three key outcomes: HHS, conversion to THA, and VAS score. For the HHS outcome (Fig 7), none of the predictors reached statistical significance. While the number of hips showed a positive trend (coefficient = 0.0598, p = 0.138), and mean age also had a positive coefficient (3.2428, p = 0.34), their 95% CI included zero, suggesting substantial uncertainty. Interestingly, disease stage had a relatively large negative coefficient (−4.925), indicating a potential association with poorer hip function, though it was not statistically significant (p = 0.287).

**Table 3:** Meta-Regression Result Summary.

Outcome Variable	Predictor	Coefficient	Standard Error	95% CI	P-value
**HHS**	Number of Hips	0.0598	0.0249	[0.01, 0.11]	0.138
	Male Percent	−0.0586	0.6196	[-1.27, 1.16]	0.933
	Mean Age	3.2428	1.9203	[-0.52, 7.01]	0.34
	Disease Stage	−4.925	3.426	[-11.64, 1.79]	0.287
**Conversion of Total Hip Arthroplasty (THA)**	Number of Hips	0.0001	0.0007	[-0.0, 0.0]	0.845
	Male Percent	0.0042	0.0055	[-0.01, 0.02]	0.48
	Mean Age	0.0094	0.009	[-0.01, 0.03]	0.347
	Disease Stage	−0.1669	0.1293	[-0.42, 0.09]	0.253
**VAS Score**	Number of Hips	−0.0171	0.0325	[-0.08, 0.05]	0.635
	Male Percent	0.0937	0.308	[-0.51, 0.7]	0.781
	Mean Age	−0.8029	0.2807	[-1.35, -0.25]	0.065
	Disease Stage	−2.7257	6.9151	[-16.28, 10.83]	0.72

For conversion to THA (Fig 8), all predictors showed minimal effect sizes and non-significant p-values, with CI consistently crossing zero. This suggests that none of the included variables—number of hips, male percent, mean age, or disease stage—had a meaningful or reliable association with the likelihood of patients requiring THA surgery. Similarly, the VAS score results (Fig 9) showed mostly non-significant associations. However, mean age emerged as a borderline predictor, with a negative coefficient (−0.8029) and a p-value of 0.065. The 95% confidence interval [−1.35, −0.25] barely excluded zero, indicating a potential trend where increased age might be associated with reduced pain intensity. This finding, although not conventionally significant, may warrant further investigation.

Overall, the meta-regression analyses indicate that the studied predictors did not have statistically significant effects on the examined outcomes, with the possible exception of mean age in relation to pain severity (VAS score). The wide CI and consistently high p-values across most models emphasize the limited explanatory power of these study-level characteristics in this context.

### Leave-one-out analysis

**Fig 10:**
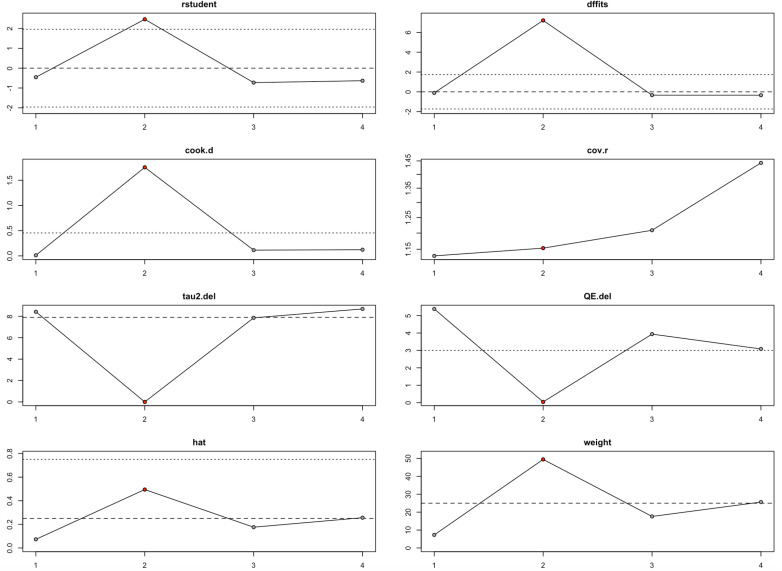
Baujat Plot for HHS.

The leave-one-out sensitivity analysis helped us better understand how individual studies influenced the overall findings for HHS, conversion to THA, and VAS pain scores. For HHS, the analysis (Fig 10, Table 4) showed that while overall heterogeneity ranged from moderate to substantial (I² between 42.68% and 61.02%), removing the second study dramatically reduced the heterogeneity to 0%. This finding, reinforced by the student, DFFITS, Cook’s distance, and hat value diagnostics, suggested that the second study had a disproportionate impact. Even so, the pooled estimates remained statistically significant across all iterations, indicating that the results for HHS were fairly robust, though somewhat sensitive to this specific study.

**Table 4:** Leave-One-Out Result Summary.

Outcome	Leave-One-Out	Estimate	SE	Z-value	P-value	CI Lower	CI Upper	Q	Qp	Tau²	I² (%)	H²
HHS	−1	8.409	2.1379	3.9333	0.0001	4.2188	12.5993	5.3818	0.0678	8.4276	61.02	2.57
	−2	5.5287	2.1598	2.5598	0.0105	1.2956	9.7618	0.0434	0.9785	0	0	1
	−3	8.8732	2.211	4.0132	0.0001	4.5397	13.2067	3.9365	0.1397	7.8581	52.9	2.12
	−4	8.897	2.4154	3.6835	0.0002	4.163	13.6311	3.0833	0.214	8.6896	42.68	1.74
Conversion to THA	−1	−1.1367	0.7029	−1.6173	0.1058	−2.5143	0.2408	17.7685	0.0001	1.2959	88.37	8.6
	−2	−0.6144	0.5501	−1.1169	0.264	−1.6926	0.4638	3.4999	0.1738	0.3922	45.68	1.84
	−3	−1.7783	0.5671	−3.136	0.0017	−2.8897	−0.6669	5.7524	0.0563	0.5443	67.1	3.04
	−4	−1.3383	0.9928	−1.348	0.1777	−3.2842	0.6075	15.7576	0.0004	2.0711	85.3	6.8
VAS Score	−1	−37.7027	32.5458	−1.1584	0.2467	−101.4914	26.086	85.0714	0	3754.218	97.34	37.6
	−2	−7.2972	47.4578	−0.1538	0.8778	−100.3128	85.7185	471.163	0	8474.18	99.66	292.31
	−3	−13.6584	47.4019	−0.2881	0.7732	−106.5643	79.2476	296.961	0	8428.026	98.67	75.28
	−4	22.6954	27.6347	0.8213	0.4115	−31.4675	76.8584	381.583	0	2672.516	99.01	100.65
	−5	−10.7807	45.0622	−0.2392	0.8109	−99.101	77.5396	489.901	0	8040.684	99.69	318.3

**Fig 11:**
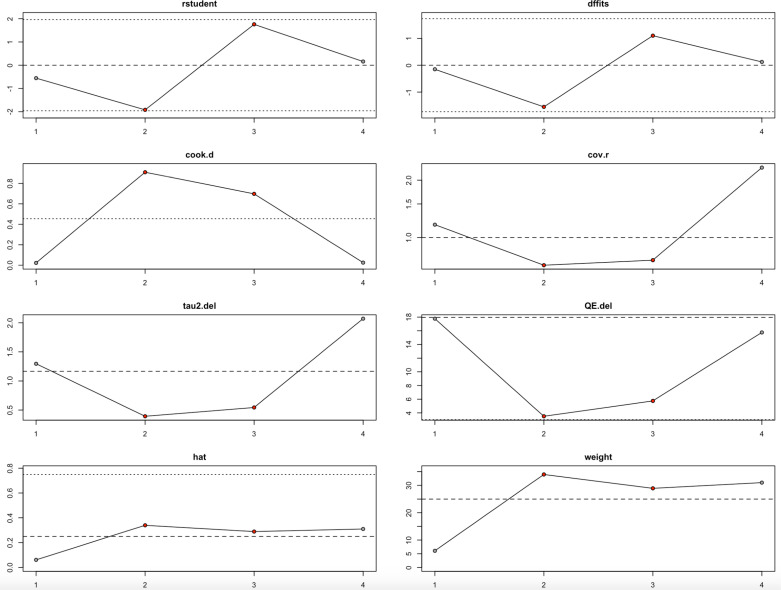
Baujat Plot for Conversion to THA.

When examining the conversion to THA outcome (Fig 11, Table 4), heterogeneity remained high regardless of which study was omitted, with I² values fluctuating between 45.68% and 88.37%. The third study appeared to exert the strongest influence, as its removal significantly shifted the effect estimate and increased influence diagnostics beyond conventional thresholds. Despite this, the overall direction of the effect did not change meaningfully, suggesting that the main findings for conversion to THA are relatively stable but still influenced by particular studies.

**Fig 12:**
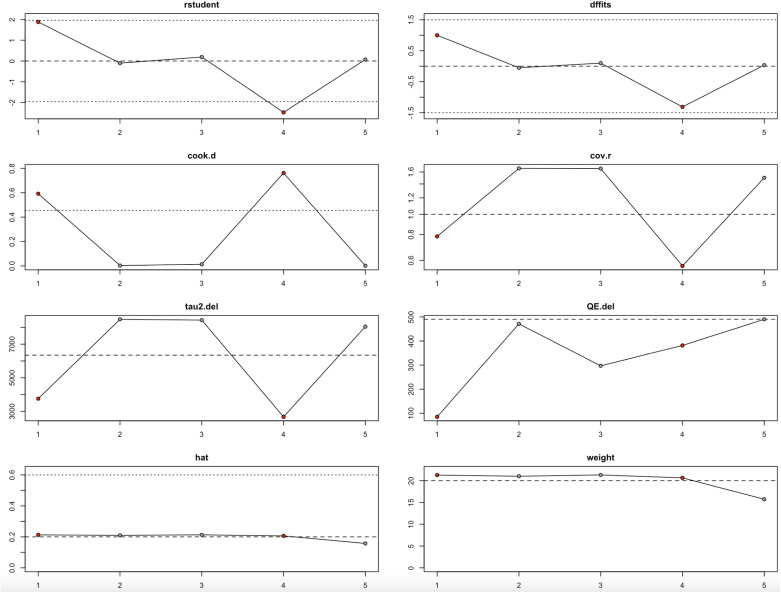
Baujat Plot for VAS Score.

The results were notably different for the VAS pain score outcome (Fig 12, Table 4). Across all iterations, heterogeneity was consistently extreme, with I² values close to or exceeding 99%, and tau² values remaining exceptionally large. Certain studies, especially the second and fourth, stood out as particularly influential, as shown by substantial changes in Cook’s distance, DFFITS, and tau² when they were omitted. However, even after excluding these studies, the results remained statistically nonsignificant, highlighting the fragility of the VAS findings. The high inconsistency and strong dependence on individual studies make these results less reliable and warrant cautious interpretation.

Altogether, the leave-one-out analysis shows that while the findings for HHS and conversion to THA are largely robust, they are still somewhat sensitive to particular influential studies (Figs 10–11, Table 4). In contrast, the VAS score results are highly unstable and heavily driven by individual studies (Fig 12, Table 4), suggesting a need for deeper exploration of potential sources of heterogeneity in future research.

## Discussion

### Heterogeneity and intervention variations

From the meta-analysis conducted on the VAS, HHS, conversion to THA, and the occurrence of femoral head collapse, the overall results reveal significant effects (p < 0.05) indicating improvements in each of these outcomes. The high heterogeneity observed in VAS (I2 = 99%) and conversion to THA (I2 = 83%) can be attributed to variations in disease stage at the time of intervention and differences in diagnostic criteria used across the included studies. Notably, one study employed a distinct intervention involving BBC implantation, which differs from the use of BMSCs in other studies [17]. BBC contains leukocytes in a concentrated suspension, originating from whole blood or bone marrow, while BMSCs are essentially multipotent stem cells [23].

### Promising effects of stem cell therapy

Our study findings suggest that stem cell therapy holds significant promise for improving outcomes in patients with AVN hip. Mesenchymal stem cells exhibit a behavior known as MSC polarization, wherein MSCs can respond to the presence or absence of inflammatory molecules in their microenvironment [10]. This has been substantiated by several studies summarized in the review conducted by Mishra VK, et al. (2020), which demonstrated that MSCs secrete TGF-beta, hepatocyte growth factor (HGF), and other factors in response to elevated levels of IFN-gamma and TNF-alpha [10]. Additionally, the promising effects observed in this study align with the results of the previous meta-analysis, which demonstrated the significance of stem cell therapy in reducing the overall occurrence of femoral head collapse (RR 0.54 [0.33; 0.89]) and the need for THA (RR 0.55 [0.34; 0.90]) [11]. This study lends substantial support to the effectiveness of stem cell therapy as a highly viable treatment option for patients with AVN hip.

### Importance of early intervention

The findings of this study also show that getting treated in the earlier stages obtains better outcomes. This statement is proven by the studies conducted by Kang, et al. (2018); Dewei, et al. (2014); Ma, et al. (2014); and Tabatabaee, et al. (2015), which compare the clinical outcome, side effects, and treatment failure which give overall better result in patients [14,16,17]. This is because in the early stage, no cortical collapse and subchondral lytic lesions have occurred yet, so stem cells can maximize the regeneration of cortical lesions in early stage of AVN. Furthermore, stem cells have the ability to self-renew and multiply, when injected into the necrotic femoral head, they can differentiate into osteoblasts, chondrocytes, and other tissues to repair dead bone [24]. In addition, stem cells can also secrete many biological factors such as various growth factors, cytokines and exosomes to promote angiogenesis and rebuild blood supply, which will reduce blood pressure.

Our findings indicate that stem cell therapy is effective in treating AVN hip, particularly in the early stages. The results show significant improvement in the HHS, reduction in VAS scores, and a decrease in the need for THA. The efficacy of stem cell therapy was most pronounced when combined with CD, which not only reduced intraosseous pressure but also introduced seed cells that facilitates the repair of the necrotic region.

### Side effects and treatment failure rates

The study also assessed the side effects and treatment failure rates associated with stem cell therapy. While the overall outcomes were positive, there were variations in effectiveness depending on the stage of AVN at the time of intervention. The earlier the stage of AVN being treated, the fewer side effects and treatment failures were observed. In advanced stages, particularly beyond stage III, the efficacy of stem cell therapy diminished, leading to higher rates of treatment failure.

### Effects of hypoxia and stromal cells on bone regeneration

Conversely, mesenchymal stromal cells exhibit the potential to exert force on bones and impede the progression of ONFH. Effects of hypoxia on osteogenic differentiation of mesenchymal stromal cells used as a cell therapy for AVN of the femoral head [25–27]. Kang et al. observed that bone marrow mononuclear cells, in conjunction with calcium phosphate, could enhance vascular endothelial growth factor (VEGF) expression, fostering osteogenesis and stimulating the regeneration of new trabecular bone [28]. Gagala et al. conducted a study revealing that the combination of BMSCs with osteochondral allografts provides structural support and promotes joint and bone regeneration [29]. Furthermore, an additional study demonstrated that BMSCs exposed to a hypoxic environment could elevate the expression levels of genes associated with bone metabolism, such as alkaline phosphatase, type I collagen, and osteocalcin, thereby stimulating bone metabolism and facilitating ONFH repair actions. Consequently, stem cell therapy emerges as a promising approach for ameliorating the progression of ONFH, emphasizing the necessity for further investigations to comprehensively elucidate its effects and treatment mechanisms [26].

### Method of stem cell therapy

The combination of CD with autologous bone marrow transplantation has emerged as the most effective technique for treating AVN hip, particularly in its early stages [30]. This approach not only relieves intraosseous pressure and removes necrotic tissue but also introduces a fresh pool of stem cells capable of differentiating into essential cells for hip function [24]. These stem cells, when injected into the necrotic femoral head, can develop into osteoblasts, chondrocytes, and other tissues, facilitating bone repair. Additionally, they secrete a range of biological substances, including cytokines and growth factors, that stimulate angiogenesis and restore blood supply, further aiding in the treatment of osteonecrosis [26].

The use of bone marrow mononuclear cells combined with calcium phosphates has been shown to enhance VEGF production and promote osteogenesis by stimulating the formation of new trabecular bone. Moreover, BMSCs exposed to a hypoxic environment can elevate the expression of genes involved in bone metabolism, thereby accelerating the repair of the osteonecrotic femoral head. This combination therapy, particularly when applied in pre-collapse stages of AVN, provides structural support and encourages bone and articular regeneration, making it a highly effective treatment option [31,32].

### Clinical implications of stem cell therapy

The clinical implications of this study suggest that stem cell therapy, particularly when combined with CD, offers a promising approach for treating AVN hip. The therapy’s ability to promote bone regeneration and restore blood supply in the early stages of AVN can potentially reduce the need for more invasive procedures such as THA. However, the variability in outcomes highlights the need for careful patient selection and timing of the intervention to maximize the benefits of this therapy. Further studies are needed to refine the application techniques and to better understand the long-term outcomes and potential risks associated with stem cell therapy in AVN hip [14,33].

### Long-term effects and duration

From a long-term perspective, cell-based therapy, particularly BMSCs implantation combined with CD, appears to offer superior durability and joint preservation compared to CD alone. In a matched case–control study, Kang et al. (2018) demonstrated that after an average follow-up of 4.28 years, the femoral head survival rate in the CD + BMSCs group was significantly higher than in the CD alone group, especially beyond the 3-year mark (p = 0.02). Additionally, the need for conversion to THA was lower in the BMSCs group (28.3%) compared to the CD group (49%) at 10 years (p = 0.031). This suggests that stem cell augmentation may extend the therapeutic window of joint function before prosthetic replacement becomes necessary [14].

These findings are corroborated by Hernigou et al. (2018), who conducted a long-term study with a mean follow-up of 25 years. The study reported that hips treated with BMSCs experienced fewer collapses (28%) compared to CD alone (72%, p < 0.0001) and showed significantly delayed onset of pain recurrence (mean 15 years vs. 5 years). The cumulative need for THA was markedly lower in the BMSCs group (24%) versus the CD group (76%, p < 0.0001), and importantly, hips initially treated with stem cells were significantly less likely to require revision or re-revision surgeries (6% vs. 53%, p = 0.0003). These results underscore the long-lasting protective effect of BMSCs therapy on joint integrity and function [15].

Similarly, Zhao et al. highlighted that the therapeutic effects of BMSCs transplantation for early-stage osteonecrosis not only included symptomatic relief but also radiographic stability over time. Their study revealed sustained improvements in hip function and reduced progression rates, aligning with earlier reports that MSC-based interventions could mitigate structural collapse and prolong joint survival. The consistent findings across studies reinforce the potential of stem cell therapies in altering the natural history of osteonecrosis, particularly in younger patients or those with precollapse-stage disease [13].

### Evaluation of side effects

Although BMSCs therapy is emerging as a promising treatment for AVN of the hip, ensuring patient safety remains a central concern. Encouragingly, the studies included in this review consistently reported a low incidence of adverse effects. Most side effects were minor and transient—such as mild pain at the injection site, low-grade fever, or temporary swelling—and typically resolved without additional treatment. Importantly, no serious complications such as infections, neoplastic changes, or immune-related events were documented. Long-term follow-up data, including the 30-year study by Hernigou et al. (2018), provide compelling evidence of the safety of BMSC therapy over time, with no major adverse outcomes linked to the intervention [15]. Similarly, Zhao et al. (2015) observed no safety concerns during a five-year follow-up in patients receiving BMSC therapy for end-stage AVN, despite the complexity of cases [16]. Lim et al. (2013) also found comparable safety profiles between stem cell-treated and non-stem cell-treated groups, further reinforcing the minimal risk associated with the therapy [34].

Meanwhile, in another study that also discusses BMSCs therapy, treatment shows promise for early-stage ONFH, though it presents several limitations and potential adverse effects. A key challenge is the lack of standardized protocols for cell preparation, leading to inconsistent cell quality and uncertain dosing efficacy [35,36]. Harvesting BMSCs from the iliac crest may cause pain, bleeding, or infection, while the specialized processing requirements increase procedural complexity and costs. Additionally, BMSCs alone cannot provide structural reinforcement, often necessitating supplementary procedures like bone grafting or CD [37].

However, it is worth noting that treatment tolerability may vary depending on disease stage, with advanced AVN sometimes showing slightly higher discomfort or reduced therapeutic benefit. Overall, these findings support that BMSC therapy is not only effective but also safe, especially when applied in the earlier stages of disease progression and within structured clinical protocols.

## Strength and limitation

This study is subject to certain limitations. Firstly, the overall quality of the evidence is characterized by heterogeneity and is deemed poor. Patients in the included studies presented with ONFH stemming from various causes, such as alcohol consumption, steroid use, and vascular disease. Despite a common trajectory leading to bone death, joint collapse, and osteoarthritis, the clinical presentation, disease progression, and response to treatment vary significantly among different etiologies. This uncontrollable variability poses a notable challenge.

Moreover, there is a potential risk of bias, although the amalgamated data indicated a positive effect of adding cell therapy alongside conventional mechanical decompression. Further research is imperative to critically evaluate the genuine effectiveness of stem cell therapy. Future RCTs studies, accounting for the diverse causes of osteonecrosis, are warranted to enhance sample homogeneity and minimize the risk of confounding bias.

Secondly, although comparative studies have reported control groups of patients undergoing CD for treatment, the techniques and procedures have not been standardized. Nevertheless, since the CD technique may be similar in both the study and control groups, the results may still contribute to characterizing trends resulting from the addition of stem cells to CD in the treatment of late-stage ONFH.

Thirdly, the processing, quality, and quantity of harvested stem cells for transplantation lack standardization, thereby contributing to the heterogeneity of the data. Additional studies and protocols should incorporate quality control measures for harvesting and implanting stem cells into necrotic lesions post-CD procedures.

Ultimately, the ten studies included in the final analysis reported different outcome measures, effectively reducing the sample size for comparisons related to various primary outcomes of interest. Nevertheless, the power analysis demonstrated sufficient statistical power for each comparison, affirming the validity of these observations. Prospective, RCTs featuring stringent inclusion and exclusion criteria, as well as standardized cell harvesting, processing, and surgical techniques, are undoubtedly essential to further elucidate the genuine effectiveness of stem cell therapy in ONFH.

## Conclusion

This study demonstrates that stem cell therapy, particularly when combined with CD, is a promising treatment for AVN of the hip, showing significant improvements in clinical outcomes like the HHS and reductions in VAS scores, while also decreasing the need for THA. The therapy was most effective in the early stages of AVN, resulting in fewer side effects and lower treatment failure rates, highlighting the importance of early intervention. However, the variability in outcomes due to differences in disease stage and intervention techniques underscores the need for careful patient selection and timing. Additionally, the combination of stem cell therapy with CD enhances bone regeneration and restores blood supply, which are vital for preventing AVN progression. While the study supports the clinical potential of this approach, further research is needed to optimize application techniques and better understand long-term outcomes, ensuring maximum therapeutic benefits for patients with AVN hip.

### Recommendation

More studies need to be conducted for further radiologic assessment with standardized scoring systems to evaluate the efficacy of the mesenchymal stem cell as a novel therapy for AVN hip.

## Supporting information

S1 Study selection. (XLSX)

S2 Data extraction. (XLSX)

S3 Risk of bias assessment. (XLSX)

S4 Prisma checklist. (DOCX)
